# Artificial Tooth Crowns

**Published:** 1891-02

**Authors:** J. B. Carmichael

**Affiliations:** Milwaukee


### THE DENTAL REGISTER.

Vol. XLV.] FEBRUARY, 1891. [No. 2.

### COMMUNICATIONS.

### Artificial Tooth Crowns.

BY J. B. CARMICHAEL, D D S , MILWAUKEE.

Read before the Northern Illinois Dental Society.

From my earliest student days this branch of our profession has engaged my attention, and I believe that the progress made in it has been unequaled in any other department of dentistry. Not more than ten years since dentists, in great numbers, considered the substituting of an artificial crown of very little importance. These same gentlemen have been compelled, "even to the very teeth and forehead of their faults to give in evidence against themselves," and adopt a principle which they ignored, and often denounced, and the demands made upon the profession in this branch of work has brought it up to its present standard of perfection.

The subject of crowning teeth might properly and intelligently be classified and discussed in the following order:

First. Importance of crowning teeth.

Second. Variety of teeth which require crowning.

Third. The most suitable crown for each case.

Fourth. The preparation for, and means of attachment.

Fifth. The patient.

It ought to appear to any ordinary thinking mind that the violation of nature's laws is neither philosophy nor business. "What God hath joined together let no man put asunder" is an injunction of Scriptures as applicable here as it is in the destinies of mankind in other respects. "Save the pieces" is the slang cry of many an ordinary individual when a accident occurs, or the wilful destruction of any thing appears imminent. Then why should the forceps or the pincers be muscularly applied to tear out the foundations of one of the greatest provisions of nature because hereditary disease, or injudicious living has produced the logical sequence; the destruction of vital functions. The pain consequent is only the cry of nature for a remedy, and a demand for immediate repair and preservation.

Crowning teeth is not a new discovery, for in the earliest days of dentistry and surgery ivory crowns were pegged on to the natural stump and made to do excellent service, and how the profession ever drifted into the practice of pulling out sound roots by the wholesale, and substituting teeth built upon a plate for purposes of mastication can only be partially explained. The tender gums, whose duty it is to moisten and nourish the original teeth, are burdened with a heavy rubber or metal attachment to which are fitted artificial molars, and made to suffer all the torture consequent on the pressure from mastication, besides the sensation created by decayed matter continually getting under the plates and keeping the mouth in a constant state of dirt and irritation, repulsive alike to the owner, and those with whom he is associated. Then the ghastly appearance of the person when those clumsy appurtenances are removed is enough, in the language of Othello, to " Frighten the isle from its propriety," repel all approaches of tenderness, and extinguish every impulse of love.

The plate experiment has been tested to its utmost bounds, and we are forced to acknowledge our mistake by the every day presence of men and women who pour out to us their tales of woe at having lost their teeth, which the skilled dentist of to-day would have repaired ; yea, restored in all their beauty, stability, and usefulness. It looks to me to be a much easier matter, and certainly more feasible and acceptable to the patient, that the mouth containing good, solid roots of teeth should have such roots prepared, and a set of suitable crowns built upon them in the most substantial and modern manner, than that they should be torn out and clumsy plates substituted. You have in those roots the foundation provided by nature for that purpose, and if the work is properly executed it will stand as a house that's built upon a rock. There are many advantages to be derived from this branch of dentistry. The fullness of the jaw will remain; if these roots remain they serve as guide posts, as foundations to build upon, and enable the dentist to replace the decayed and broken down teeth by a set of crowns in shape, size, symmetry, and contour like those preceding them, presenting all the stability and beauty of appearance of the natural product tending to the peace, health, and comfort of the patient, and the pleasure and satisfaction of the operator.

Having expressed ourselves on the necessity of crowning teeth, and the superiority of such over the discarded and abominable plate, we shall now endeavor to present to you, in brief, our opinion as to the various kinds of teeth that should be crowned, which is our

Second Proposition. On this subject a great variety of work appears requiring all the judgment and acute perception which natural ability and experience brings.

Should we not be thoroughly converted to the idea of crowning all injured teeth, then we should be able to accurately determine what teeth to crown, and what teeth to fill. As with the plate, the use of which has been carried to such an extreme as to make it an abuse; so, I say, with regard to building down, or building up teeth, as you please to call it. The mania for filling all teeth indiscriminately has passed into history to a great extent never, I hope, to be revived. The thought of rival dentists and ambitious novices malleting for days and weeks, perhaps, just to see how much adhesive gold could be pounded into a cavity, so as to make a walking advertisement of the patient seems equally as reprehensible as the extremes gone to in the case of the discarded plate. I have sat for four hours at a time under the operation, my powers of endurance being taxed to the utmost for the purpose of having one tooth filled, which it would have been much easier to crown, less tedious, less painful, and more durable. Therefore, my own experience both as dentist and patient, forces me to discountenance the tedious and torturing process of making these large and complicated fillings, especially in front teeth, where appearance is of the utmost importance. The remaining frail portion of badly decayed teeth, especially where the nerve has been destroyed, is not sufficient to sustain an operation of this kind, and make it of lasting utility. The amount of malleting required will often prove injurious to nervous persons, a result which could be avoided by cutting off the diseased portion, and fitting a crown in its place. In cases, however, where the nerve is alive, the teeth extremely soft and sensitive, and where it is not prudent to cut away and crown on account of the living nerve, I would make a partial crown, as follows: Swedge a piece of pure gold to the shape of the decayed portion, and covering the inside cusp. It is then placed upon the tooth and finished flush with the edges of the cavity; an impression taken in wax, getting the accurate acclusion and articulation in conformity with the natural teeth, and the destroyed portion restored. Our next and

THIRD PROPOSITION, is to discover, if possible, the most suitable crown to be used. It is not the purpose of this paper to enter into a lengthy description of the methods of making and substituting artificial crowns, nor of recommending any one crown manufactured. The details can best be understood by clinical demonstration. It is necessary, however, to make some suggestions and explanations. The crowns which come ready for use have their merits and demerits, but can not by any possibility be made to answer the purpose in the endless variety of shapes of teeth, and phases of decomposition of roots; so it remains with the dentist to reconstruct them, or to manufacture his own crowns, as getting that which will suit his purpose from the manufactured crowns is merely an accident. We will dispose then, of the manufactured crown by relegating the whole line of them to that bourne "from whence no traveler returns," as impracticable for general use, insubstantial, and no remedy for persistent decay, but merely an easy means of covering up the incompetency of the dentist. While I do not approve of any excessive use of gold in general operations, especially where prominently apparent, yet I would recommend that all posterior teeth which are used for mastication, should be fitted with a gold crown on account of its strength and durability, admitting as it does of the proper acclusion, or articulation.

Some ten years ago I introduced a crown die, the first in use as far as I know, by the aid of which perfectly shaped gold teeth can be formed either from a solid nugget, or from plate gold, thus symplifying the manufacture of crowns and bringing it within the experience and practice of every dentist. The porcelain crown, or the porcelain-faced crown, with a gold attachment covering the entire end, or exposed portion of the root, is the best crown with or without the gold band, accomplishing the most desired results on all anterior teeth. My conclusion then, is that the gold crown is the most preferable for all teeth where stability and strength are demanded because it can be struck up or moulded into any shape to suit the dentist's desire, or the requirements of any tooth. Should a whole gold tooth be required it can be moulded by means of the cuttle fish bone, a means suggested by necessity and applied by me ten years ago in dentistry. But in every case where appearance is a desideratum the porcelain-faced crown should be used as more in harmony with natural appearances in all anterior teeth, and extended also to all back teeth which are visible, or partially so. Our

Fourth Point was preparations for, and means of attachment. It is not necessary to say much on the preparation of roots; every dentist understanding it; except to suggest that we bear in mind that the more of the root you can leave above the gum margin, the easier will be your operation. You will also be careful to have the roots dressed so evenly that the gold band will fit it as closely as can be done, leaving no aperture where matter can lodge. Again, we must be careful to leave a proper space between the teeth so that the gold bands on two similar teeth, in close proximity, will not crowd each other or crowd the gum thus producing irritation and pain. The gold band used as a means of attachment is very generally accepted as the best. In using a gold band then, I should prefer thin, soft gold about 23 carats fine and 33 Stubb's gauge in thickness, being much more easily burnished to the irregularities of the root, and causing little or no irritation to the gums. In dressing the root for the crown make a free and liberal separation between the roots, avoiding any possibility of crowding the gums from between the teeth. Do not force the band unnecessarily, or press it to a depth beyond the gum margin as no strength to the crown is gained thereby, and it only produces inflammation and sloughing of the gums, and pain to the patient. Some five or six years ago I was called upon to substitute crowns upon the six anterior superior teeth for a highly nervous and sensitive lady patient who would not submit to the operation of filling a single tooth.

After cutting off the natural decayed crown I set about the task of substituting a good and permanent crown without submitting my patient to the operation of banding the root, and taking a new departure I succeeded in making a crown which has proved to be, in my experience, a decided success, avoiding giving pain to my patient, and being perfect in appearance. The attachment to this crown is made by inserting a double tube pivot in the root, a golden collar about the palatine portion, and a pure gold plate burnished over the end of the root, then an impression taken. I then proceed in the usual manner to adjust the porcelain teeth.

This crown differs from the Richmond in two important points, viz.: It has a second pivot larger than the first and encircling it, and instead of a band I extended the gold basis back so as to form a collar grasping the anterior portion of the natural root and making an immovable foundation on which to solder and solidify the superstructure of a porcelain face and a solid gold back, completing a tooth where all the metals are visible and the natural appearance is preserved. Our

Fifth Proposition: The patient shall not occupy our time except for a few remarks and then we shall close this paper. Your patient places implicit confidence in your abilily and knowledge of your art and gives you to understand that money consideration shall not stand in the way of a perfect piece of workmanship. He or she will also be reasonable enough to know that there is in dentistry, as in medicine and surgery, a limit to all human means and appliances. The prominent desire being to have the teeth put in the best possible condition and to insure to them those pleasures which can only be derived from a perfect condition of those important organs which portain to the necessities of human existence. The dentist, on his part, must exercise great patience and constant consideration for the feelings of his patient, and cultivate harmony by gentlemanly and kindly demeanor, thus bringing about mutual reciprocal sympathy, facilitating the production of a nice and lasting piece of mechanical work which shall prove to be to him a lasting evidence of his skill in a noble profession and a source of gratification and delight to the individual who selected him for the duty.

I now say "Good-by" for the present, trusting that my remarks may tend to draw out the thoughts of others, and to advance and elevate one of the grandest professions of modern civilization for the alleviation of human suffering, and the repairment and maintenance of the chief machinery of human life.

